# Dissecting a Hidden Gene Duplication: The *Arabidopsis thaliana SEC10* Locus

**DOI:** 10.1371/journal.pone.0094077

**Published:** 2014-04-11

**Authors:** Nemanja Vukašinović, Fatima Cvrčková, Marek Eliáš, Rex Cole, John E. Fowler, Viktor Žárský, Lukáš Synek

**Affiliations:** 1 Institute of Experimental Botany, Academy of Sciences of the Czech Republic, Prague, Czech Republic; 2 Department of Experimental Plant Biology, Faculty of Science, Charles University in Prague, Prague, Czech Republic; 3 Department of Botany, Faculty of Science, Charles University in Prague, Prague, Czech Republic; 4 Department of Biology and Ecology, Faculty of Science, University of Ostrava, Ostrava, Czech Republic; 5 Department of Botany and Plant Pathology and Center for Genome Research and Biocomputing, Oregon State University, Corvallis, Oregon, United States of America; University of Lausanne, Switzerland

## Abstract

Repetitive sequences present a challenge for genome sequence assembly, and highly similar segmental duplications may disappear from assembled genome sequences. Having found a surprising lack of observable phenotypic deviations and non-Mendelian segregation in *Arabidopsis thaliana* mutants in *SEC10*, a gene encoding a core subunit of the exocyst tethering complex, we examined whether this could be explained by a hidden gene duplication. Re-sequencing and manual assembly of the *Arabidopsis thaliana SEC10* (At5g12370) locus revealed that this locus, comprising a single gene in the reference genome assembly, indeed contains two paralogous genes in tandem, *SEC10a* and *SEC10b*, and that a sequence segment of 7 kb in length is missing from the reference genome sequence. Differences between the two paralogs are concentrated in non-coding regions, while the predicted protein sequences exhibit 99% identity, differing only by substitution of five amino acid residues and an indel of four residues. Both *SEC10* genes are expressed, although varying transcript levels suggest differential regulation. Homozygous T-DNA insertion mutants in either paralog exhibit a wild-type phenotype, consistent with proposed extensive functional redundancy of the two genes. By these observations we demonstrate that recently duplicated genes may remain hidden even in well-characterized genomes, such as that of *A. thaliana*. Moreover, we show that the use of the existing *A. thaliana* reference genome sequence as a guide for sequence assembly of new *Arabidopsis* accessions or related species has at least in some cases led to error propagation.

## Introduction

Evolution of plant genomes frequently involves segmental and even whole-genome duplication events. Gene duplications provide a crucial source of raw material for evolution of organisms [1]. Upon fixation, the evolutionary fate of gene duplications can follow a few different scenarios: conservation of gene function, pseudogenization, subfunctionalization or neofunctionalization (reviewed in [2]–[4]). The fate of duplicated genes, resulting from an interplay of chance and selection, appears to correlate with their function, as well as their mode or history of duplications. Gene duplicates within certain functional categories are retained or lost with varying probability in flowering plants [5], [6], and products of whole genome duplications behave differently from those resulting from single gene tandem duplications [7].

The nuclear genome of *Arabidopsis thaliana*, one of the smallest genomes among land plants, and undoubtedly the best characterized one, contains over 27,400 protein-coding genes (see TAIR – http://www.arabidopsis.org and [8]) and exhibits a significantly higher fraction (37%) of predicted genes belonging to gene families with more than five members, compared to organisms with a similar number of genes – *Drosophila melanogaster* (12%) or *Caenorhabditis elegans* (24%), reflecting more abundant gene duplications [9]. This phenomenon may be explained, e.g., by more relaxed constraints on the genome size in plants, by a more prominent role of unequal crossing-over to generate new gene copies [9], or by a selective advantage of subtle functional tuning, or subfunctionalization, contributing to the survival of paralogous genes in sessile organisms as adaptation to unavoidable occasional adversity of local conditions [3].

The *Arabidopsis thaliana* genome sequencing project engaged the strategy of hierarchical or clone-by-clone sequencing [9]. In essence, the genome was first broken into large fragments that were subsequently cloned into BACs (Bacterial Artificial Chromosomes) in order to obtain a genomic library. Afterwards, each BAC was read using the shotgun sequencing method, employing another round of fragmentation and Sanger sequencing. All reads were then computationally assembled to create contiguous sequences corresponding to BACs and to original chromosomes. The assembly step has long been known as a possible source of errors in genome sequence reconstruction, especially in the case of highly identical repetitive sequences longer than an average read length [10], [11]. While this problem has been recognized in human and rodent genome assemblies [12]–[15] and partly overcome by more advanced algorithms, such as ARACHNE [16] or PCAP [17], as well as by “next generation” assembly algorithms developed in parallel with the new high-throughput mass parallel sequencing techniques (reviewed in [18]), the bulk of the current “gold standard” *A. thaliana* genome assembly predates these methodological improvements. While occasional corrections are being introduced regularly in the process of genome updating and re-annotation, they currently, as a rule, concern only point mutations and short (several bp) indels spanned by cDNA or EST (Expressed Sequence Tag) sequences [19].

The availability of *A. thaliana* genome sequence opened a gate towards complete inventories of evolutionarily conserved genes. We have previously used sequence information to find homologs of all subunits of the exocyst complex in *Arabidopsis* [20]–[23]. This hetero-octameric protein complex, consisting of Sec3, Sec5, Sec6, Sec8, Sec10, Sec15, Exo70, and Exo84 subunits, functions in the last steps of exocytosis – in docking and tethering of secretory vesicles to the plasma membrane (reviewed in [24], [25]). Genes encoding all eight exocyst subunits were also found in all land plants examined, often forming families of paralogs, which is in contrast to the situation in yeast or metazoan, where each subunit is encoded by a single gene or (in vertebrates) small families of paralogs [23]. *A. thaliana* has two SEC3, SEC5 and SEC15 paralogs, three EXO84 paralogs, and 23 EXO70 paralogs, a number unparalleled outside the plant kingdom [21]–[23], [26]. The remaining three subunits, SEC6, SEC8, and SEC10, are each encoded by a single gene according to the current genome annotation.

Here, we report that the SEC10 exocyst subunit in *A. thaliana* is in fact encoded by two genes in tandem, and that 7 kb of sequence at the *SEC10* locus (At5g12370) is missing in the *A. thaliana* reference genome assembly. This demonstrates that assembly errors involving highly similar sequences in tandem duplication may lead to genome sequence artifacts and omission of functional genes even in a well characterized genome such as that of *A. thaliana*. We also document here that the two *SEC10* genes are most probably functionally redundant in plant cells.

## Results

### Different lines of evidence hint for the presence of two copies of the *SEC10* gene in the *A. thaliana* genome

A single gene (At5g12370) coding for the SEC10 exocyst subunit was previously identified in the genome of *Arabidopsis thaliana* [21]. However, two independent lines of evidence led us to suspect that the At5g12370 locus may have been incorrectly assembled during the genome sequencing due to a tandem gene duplication, and that at least two genes encoding SEC10 are present in the *A. thaliana* genome.

First, a *SEC10* cDNA sequence [GenBank: AF479280] that we obtained by sequencing a cDNA clone identified on the basis of partial EST sequence data [GenBank: AV528809] [27] exhibited multiple single nucleotide mismatches compared to any of the alternative reference (TAIR10) *SEC10* cDNA sequences predicted on the basis of genomic data [GenBank: NM_121275.4]; alternative predictions [GenBank: NM_001036794.1; GenBank: NM_001036795.2] are not considered further for simplicity. The same discrepancy with the reference sequence was also evident in a cDNA sequence [GenBank: AY096638] originating from a large-scale cDNA sequencing project [28]. The coding sequences of AF479280 and AY096638 are identical (except a single substitution in AY096638 most likely reflecting a sequencing error), and are furthermore identical to the coding sequence of a *SEC10* gene that we previously obtained by RT-PCR (Reverse Transcription PCR) from *A. thaliana* Col-0 seedlings [29]. Parts of the untranslated regions (UTR) flanking the coding sequences of AF479280 and AY096638 are also identical, although the very 5′ and 3′ extremities of the two cDNAs differ (see below). When compared to the reference cDNA sequence (NM_121275.4), both AF479280 and AY096638 exhibit 27 single nucleotide differences in 12 out of 24 coding exons, whereas the remaining coding exons are identical (overall sequence identity within the coding sequence is thus over 99%). In addition, an extra 12-bp-long sequence is present within exon number 16 of AF479280 and AY096638, and is without a counterpart in the reference genome sequence. On the other hand, using BLAST, we found another *SEC10* cDNA [DDBJ: AK222187] from a large-scale cDNA sequencing project [30], which does match perfectly the reference cDNA sequence [GenBank: NM_121275] except for the last 72 bp of the 3′ UTR, which do not align with the reference cDNA at all and have no corresponding sequence even in the genome assembly, raising thus the possibility of a cloning artifact.

Second, in several *Arabidopsis* mutant lines with T-DNA insertions in *SEC10*, the offspring of self-crossed putative heterozygous plants, which were fully fertile, exhibited a confusing segregation ratio of 1∶3∶0 or 0∶1∶0 (w/w ∶ w/m ∶ m/m) when analyzed by PCR genotyping (Table S1). This could not be explained by embryonic or gametophytic lethality, and therefore, we speculated that a PCR product corresponding to the wild-type allele might be amplified from another (yet unknown) very similar *SEC10* paralog present in the genome. We proposed that the apparent heterozygotes in the populations segregating were, in fact, a mixture of genuine heterozygotes and homozygotes that exhibited a wild-type signal from another *SEC10* paralog in PCR genotyping. In the latter case (0∶1∶0), the assumed heterozygous parent plants were most likely homozygous plants in fact. If this was the case, and homozygous plants were present in the offspring, no obvious mutant phenotype was noticed. This is notably unlike mutants in the two other exocyst subunits encoded by a single gene, as *SEC6* and *SEC8* exhibit pollen-specific transmission defects of mutant alleles [29], [31]. This suggested that the function of the disrupted gene might be complemented by an unknown second gene also encoding a SEC10 subunit of the exocyst complex.

### Re-sequencing of the *SEC10* locus reveals the presence of tandemly duplicated *SEC10* genes

Few gaps are known to remain in the *A. thaliana* reference genome sequence, most of them in centromeres and pericentromeres (http://www.arabidopsis.org/portals/genAnnotation/gene_structural_annotation/agicomplete.jsp). If the hypothetical second *SEC10* copy does not reside in a gap, it would be most likely located at the *SEC10* locus itself, and its absence from the reference genome sequence may be due to an assembly artifact caused by collapsing a tandem duplication of the *SEC10* gene into one copy.

**Figure 1:**
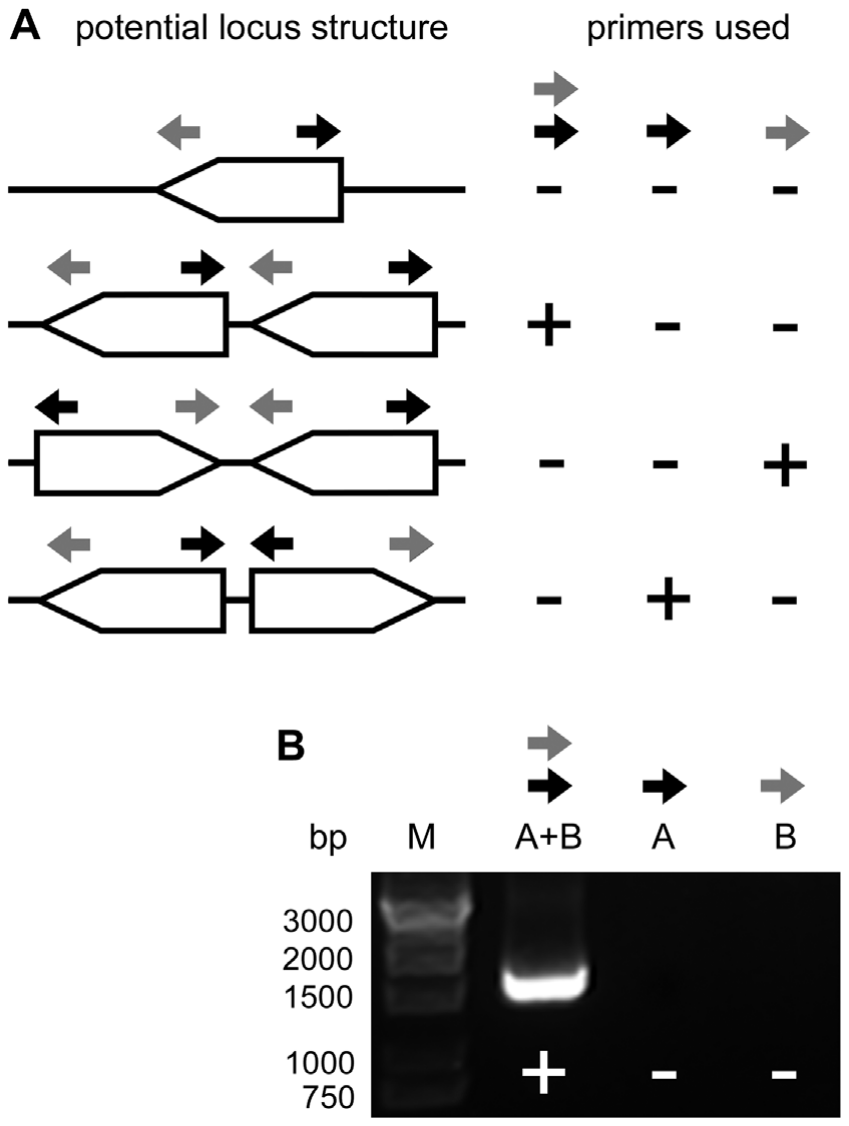
Evidence for *SEC10* gene tandem duplication. (**A**) Expected outcomes of diagnostic PCR with outward-facing primers “A” and “B” (gray and black arrows), specific to each end of the *SEC10* gene, for potential structures of the *SEC10* locus (a single gene or three variants of tandem gene duplication). The table on the right shows the expected presence or absence (+ or −) of PCR products using different primer combinations. (**B**) Results of PCR reactions according to (**A**) using A. thaliana Col-0 genomic DNA as a template.

**Figure 2:**
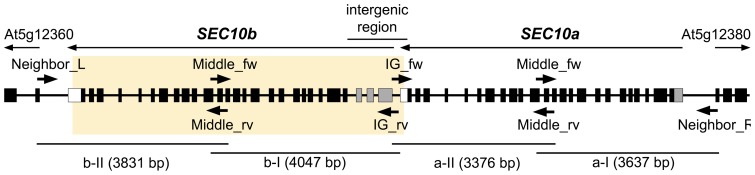
The revisited structure of the *SEC10* locus in *Arabidopsis thaliana*. The revisited arrangement of the *SEC10* locus (At5g12370) depicts *SEC10a*, *SEC10b*, and parts of two neighboring genes (At5g12360, At5g12380). Coding exons are shown as black boxed, 5′UTR as gray boxes, and 3′ UTR as white boxes. Arrows indicate the position and orientation of primers used for cloning of the *SEC10* locus in four overlapping parts (a-I, a-II, b-I and b-II; lines at the bottom represent the ranges of the cloned PCR products). The orange strip marks the region omitted from the reference sequence of the *A. thaliana* genome.

To test this hypothesis, we designed a pair of outward-facing PCR primers, A and B (Figure 1A; Table S2), matching the first and the last exon, respectively, of the *SEC10* gene in regions that are identical in the two different *SEC10* cDNA versions. Depending on the presence and orientation of another *SEC10* version(s), PCR reactions using primer A only, primer B only, or both primers together on genomic DNA template should yield products allowing us to distinguish between the possible locus arrangements (Figure 1A). Indeed, using genomic DNA from the Columbia-0 (Col-0) ecotype as a template, we obtained a PCR product only with the combination of A and B primers. This product corresponds to the presumed intergenic region between the hypothetical tandemly duplicated *SEC10* genes in the “head to tail” orientation (Figure 1B). The same product was obtained also on templates from two other *A. thaliana* ecotypes, Landsberg erecta (Ler-0) and Nossen (No-0) (Figure S1A), indicating that the gene duplication is not restricted to the Col-0 ecotype. In contrast, a similar experiment with *Arabidopsis lyrata* using species-specific primers showed no duplication in this species (Figure S1B). To distinguish the two *SEC10* genes in *A. thaliana*, we labeled the upstream gene (in the direction of transcription) as *SEC10a* and the downstream gene as *SEC10b* (Figure 2).

The PCR product obtained from Col-0 DNA was cloned and sequenced, providing an intergenic sequence of 1511 bp between the stop codon of *SEC10a* and the start codon of *SEC10b*. The first 108 nucleotides at the 5′ end of the intergenic sequence (i.e. downstream of the stop codon of the *SEC10a* gene) were identical to the region of the reference genome sequence immediately downstream of the sole *SEC10* gene (*A. thaliana* chromosome 5 [GenBank: CP002688.1], positions 4002894-4003001 in the complementary strand), but the rest of the amplified segment could not be matched perfectly to the reference genome sequence (Data S1). Thus, nearly the complete intergenic region between the *SEC10a* and *SEC10b* genes, and the whole coding sequence of the *SEC10b* gene, are missing from the current reference genome sequence of *A. thaliana*.

Using the newly determined intergenic sequence and sequences of the genes flanking the *SEC10* locus, we designed specific primers to amplify and clone both *SEC10* versions present in the *A. thaliana* Col-0 genome. As we were unable to amplify whole *SEC10* genes, probably due to their length of approx. 8 kb, we cloned each *SEC10* gene in two halves using additional primers designed on the basis of the known cDNA sequences of both SEC10 versions and matching internal exons conserved between the two *SEC10* genes (Figure 2). Four overlapping PCR products covering the entire *SEC10* locus were obtained, cloned and sequenced. Finally, a complete sequence of the *SEC10* locus was manually assembled [ENA: HG764169].

Comparison of our assembled sequence, including the *SEC10* tandem duplication, with the reference genome sequence revealed that a 7 kb sequence segment is missing from the reference (Figure S2). Because the artificial deletion occurs in a duplicated region, its position with respect to the current sequence of the chromosome 5 [GenBank: CP002688.1] cannot be unambiguously defined. We arbitrarily define the position of the deletion between the nucleotides 4002893 and 4002894 of the current assembly of the chromosome 5, in which case the deleted region comprises (in the direction identical with the orientation of the two *SEC10* genes) a part of the 3′ UTR region of the *SEC10a* (corresponding to the AK222187 cDNA, see above), the putative promoter region of the *SEC10b* gene, and nearly the whole *SEC10b* itself, except the very terminus of its 3′ UTR (Figure 2 and S2). This means that the *SEC10* gene sequence in the reference genome assembly is a chimera consisting primarily from *SEC10a*, except a region in its 3′ UTR derived from *SEC10b*. We therefore suggest that the systematic gene ID assigned to the original misassembled *SEC10* gene, At5g12370,should be used to designate the *SEC10a* gene, whereas the *SEC10b* gene could be designated with a new ID, At5g12365.

A comparison of the *SEC10a* and *SEC10b* sequences (their alignment in Data S1) revealed regions of discernible homology both upstream of the CDS (including a putative promoter and the transcribed 5′ UTR) and downstream of the CDS (the 3′ UTR and a region downstream of the polyadenylation site). The first fifth and the last sixth of the sequence between the start and stop codons is identical with an exception of four substitutions. There are at least 40 indels between the two paralogs, ranging from 1 to 35 nucleotides. All but one are located in the non-coding regions. The indel in the coding sequence occurs in the 16^th^ coding exon and accounts for twelve nucleotides. The identity of coding exons ranges between 95% and 100%, whereas the identity of introns ranges between 79% and 100% (ignoring indels longer than one nucleotide). A comparison of the *A. thaliana SEC10* genes with the sole *A. lyrata* homolog revealed that At*SEC10a* and At*SEC10b* are mutually more similar than any of them to *A. lyrata SEC10* (Data S2), suggesting that the duplication occurred after the divergence of the *A. lyrata* and *A. thaliana* lineages. This comparison also suggests that the twelve-nucleotide indel in the coding sequences of *SEC10a* and *SEC10b* is due to a deletion in *SEC10a* rather than an insertion in *SEC10b*.

Differences in the predicted protein sequences of the two *SEC10* paralogs are minor, since most of the substitutions in exons are silent. The SEC10a and SEC10b proteins differ only by substitution of five amino acid residues (G4R, A235T, V500F, D503E, T679P), in addition to a deletion of four amino acid residues (TSVS at position 569) in the SEC10a protein (Figure S3). This high degree of similarity suggests that SEC10a and SEC10b isoforms might be functionally redundant.

### Both *SEC10* gene copies are expressed in *A. thaliana*, yet *SEC10b* is the dominant isoform

Using our complete sequence of the *SEC10* locus, we could explain the differences in the various *SEC10* cDNA sequences obtained by us or others (see above and Table S2). Thus, the cDNA AK222187 [30] could be unambiguously assigned to *SEC10a*, whereas the cDNAs AF479280 and AY096638 [28] match perfectly the *SEC10b* gene. The differences in the 5′ and 3′ UTRs of AF479280 and AY096638 most likely result from an alternative transcription initiation and polyadenylation, with the transcription start for the AY096638 sequence located within the region corresponding to the second intron as defined by the AF479280 sequence and with polyadenylation starting downstream of that in AF479280.

Earlier, we amplified the coding sequence of the *SEC10b* cDNA using a total cDNA prepared from Col-0 seedlings [29]. However, repeated attempts to amplify the coding sequence of *SEC10a* from cDNA templates prepared from various tissues and stages of Col-0 using primers that would amplify both *SEC10a* and *SEC10b* yielded only additional *SEC10b* clones (40 clones tested in total), as determined by restriction analysis of coding sequences amplified from the clones (Figure S4A). This suggests low expression of the *SEC10a* gene, albeit the existence of the AK222187 cDNA [30] proves that it indeed is expressed. We then used cDNA prepared from *sec10b-1* homozygous T-DNA insertional mutants (SALK_120710) as a template. Two types of PCR products of slightly different size were cloned (Figure S4B). The longer ones were identified as *SEC10a* by restriction analysis, and sequencing of two independent clones showed that they match exactly the predicted coding sequence of the *SEC10a* cDNA. Sequencing of the shorter products revealed that they were out-of-frame deletion derivatives of the *SEC10b* cDNA, presumably non-functional, containing most of the region downstream of its T-DNA insertion site. This aberration may have arisen by transcribing *SEC10b* with its T-DNA insertion, which was then spliced out together with the whole second and a part of the first exon. Splicing out T-DNA insertions has been reported before [32], [33], albeit it may be a rare event.

**Figure 3:**
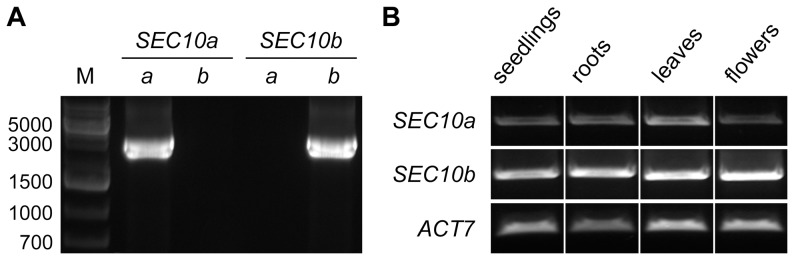
Expression levels of *SEC10a* and *SEC10b i*n various tissues of *A. thaliana*. (**A**) Specifity of the PCR primers demonstrated on paralog-specific cDNAs (AK222187 for *SEC10a* and AY096638 for *SEC10b*; indicated above the line), using primer sets specific for *SEC10a* or *SEC10b* (indicated below the line as *a* or *b*, respectively). (**B**) Expression levels of *SEC10a* and *SEC10b* in various tissues as analyzed by semi-quantitative RT-PCR. The expression level of the *ACT7* gene was used as a control.

To analyze the level of expression of each isoform we designed two unique sets of primers, which can reliably discriminate between *SEC10a* and *SEC10b* (Figure 3A). Expression of *SEC10* genes was analyzed by semi-quantitative RT-PCR on four different total cDNAs prepared from young seedlings, roots, leaves and flowers, respectively. In all cases, *SEC10b* appeared to be a dominant isoform, showing higher expression in all samples (Figure 3B), which is in good agreement with the previously observed higher frequency of *SEC10b* clones in wild-type plants. We conclude that both *SEC10a* and *SEC10b* are functional genes that are expressed in *A. thaliana*.

### Analysis of insertional mutants indicates a functional redundancy of *SEC10a* and *SEC10b*

**Figure 4:**
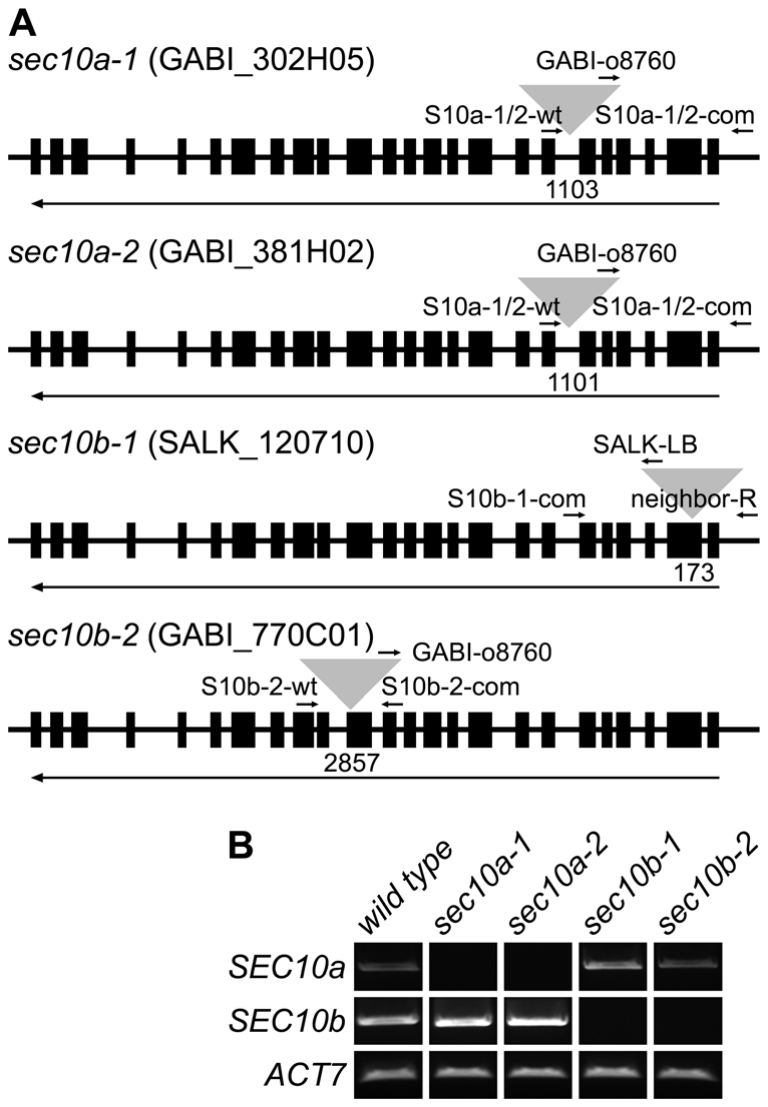
Analysis of T-DNA insertional mutants in *SEC10* genes of *A. thaliana.* (**A**) Positions of T-DNA insertions and primers used for genotyping (Table 1) are indicated by triangles or arrows, respectively. Numbers below genes indicate the exact position of each insertion (in bp counted from the start codon) and long arrows show the gene orientation. (**B**) Expression levels of *SEC10a* and *SEC10b* in young seedlings of mutant lines as analyzed by semi-quantitative RT-PCR. The expression level of the ACT7 gene was used as a control.

Using the revised sequence of the *SEC10* locus, we designed paralog-specific sets of primers for PCR genotyping and performed new segregation analyses of selected T-DNA insertional mutant lines in both *SEC10a* and *SEC10b* (Figure 4A). For each line, we performed sequencing of the region flanking the Left border of the T-DNA to determine whether the T-DNA is inserted in *SEC10a* or *SEC10b* (flanking sequences provided by the collections were usually insufficiently long with respect to high similarity between *SEC10a* and *SEC10b*). Semi-quantitative RT-PCR showed that all mutant lines are null alleles, expressing no detectable specific mRNA; whereas transcripts from the unaffected paralog were detected in all cases (Figure 4B). The segregation ratio in all mutant lines was compatible with Mendelian rules (1∶2∶1) (Table 1) and a phenotype analysis of *sec10a* and *sec10b* homozygous mutants revealed no observable deviations from wild-type plants. Thus, we suggest that both genes share overlapping functions and exhibit redundancy under standard culture conditions.

**Table 1:** Insertional mutations in the *A. thaliana SEC10* locus do not affect viability.

Mutant line	Mutant allele	T-DNA position	Segregation ratio w/w:w/m:m/m	Statistical evaluation*	
				?^2^	P
*sec10a-1*	GABI_381H02	intron 6	22:62:30	2.000	0.368
*sec10a-2*	GABI_302H05	intron 6	32:66:29	0.339	0.844
*sec10b-1*	SALK_120710	exon 2	30:63:41	2.284	0.319
*sec10b-2*	GABI_770C01	exon 14	42:67:30	2.252	0.324

* Testing a difference from normal segregation ratio 1:2:1 using the Chi-square test.

## Discussion

Repetitive sequences, including gene duplications, present a major source of computational difficulties for genome sequence assembly and mapping based on shotgun sequencing approaches. Serious errors in the reference genome sequence of rice caused by assembling of repetitive sequences were identified recently [34]. Importantly, comparison of two independent assemblies of the human genome sequence based on clone-by-clone sequencing or whole-genome shotgun sequencing (WGS), respectively, revealed that at 95.5% or greater sequence identity large segmental duplications disappear from the WGS assembly [14]. It is estimated that 50%–60% of highly similar (>90%) segmental duplications are not resolved as duplicated copies within the WGS assemblies of human, mouse and rat genome sequences [35]. At >97% identity, the portion of unresolved duplications increases up to 91% as calculated in She *et al*. [14]. For example, single-nucleotide polymorphisms interpreted in databases as different alleles could often be potential paralogous sequence variants, depending on the threshold set in the assembling software [13]. Assembling the shotgun reads from individual clones eases the task because duplications are often split into different non-overlapping clones. Therefore, the clone-by-clone sequencing approach is superior to WGS in the resolution of segmental duplications [14]. Although clone-by-clone sequencing was employed in the *A. thaliana* genome sequencing project [9], the computational sequence assembly of individual clones (BACs, ∼200 kb) is still largely sensitive to within-clone near-identical segmental duplications (especially when in tandem), albeit the complexity of sequences assembled from shotgun data is relatively low. Reassembling of the *Arabidopsis* genome sequence from the original reads using the latest software would be advisable to uncover at least a portion of hidden gene duplications. Alternatively, remapping of the original reads to the genomic sequence assembly and subsequent analysis of regions that exhibit an excessive read coverage may detect sites of potential duplication that would be further inspected manually [14], [36].

In case of the *A. thaliana SEC10* gene, the history of reference sequence updates did not suggest an assembly problem. Although the whole locus was missing (i.e. located within a gap) in the original genome sequence release ([9], GenBank: NC_003076_1), its reference sequence underwent no changes since it appeared in the first revision of the Chromosome V reference sequence in 2001 (GenBank: NC_003076_2). However, we noticed the existence of cDNA sequences incompatible with the reference genome sequence (already mentioned in [23]), and obtained suspicious results from genotyping insertional mutants in the *SEC10* gene, which prompted us to re-sequence the whole *SEC10* locus. Since only one PCR product was amplified in a reaction employing outward-facing primers (Figure 1), we concluded that a single tandem repeat of *SEC10* gene is probably present in the genome (unless the intergenic regions between the potential additional *SEC10* copies have an identical length). Subsequent subcloning and sequencing with manual assembly revealed that the locus indeed harbours two copies of the *SEC10* gene, similar enough to be collapsed into one chimeric locus by assembly algorithms. As a result, 7 kb were omitted from the final genome sequence, apparently due to an error during the sequence assembly of the BAC clone T2L20 [ENA: AL592312.1] that represents the region including the *SEC10* locus in the *A. thaliana* chromosome 5 pseudomolecule. We attempted to obtain the original raw sequencing reads for the T2L20 clone, but unfortunately they have not been retained after the completion of the *Arabidopsis* genome project (Mike Bevan, John Innes Centre, Norwich, UK, personal communication), so we could not directly revisit the assembly of the T2L20 sequence and had to employ the strategy based on PCR amplification of the misassembled *SEC10* locus.

Since the *A. thaliana* reference genome sequence has been used as a framework for sequence assembly in numerous additional sequencing projects aimed at characterizing *Arabidopsis* genome diversity, the omission of one of the two *SEC10* genes may have been propagated into additional sequencing projects. In particular, the Ler-0 genome sequence [37], as well as Bur-0, C24 and Kro-0 ecotypes accessible at the website (http://www.1001genomes.org) of the “1001 genomes” project [38], contains a single *SEC10* gene, even though our PCR-based test indicates the presence of two *SEC10* copies in Ler-0, similar to the Col-0 ecotype. However, somewhat encouragingly, the SEC10 protein prediction is missing in several of the 19 predicted proteomes derived from the first phase of the “1001 genomes” study (available at http://mus.well.ox.ac.uk/19genomes/), including No-0, another accession shown here to carry the duplication. Thus, although the error propagation problem in reference-guided genome assemblies undoubtedly exists, it might be to some extent self-limiting, since gene duplications may result in assembly problems leading to exclusion of problematic sequence areas from further processing. Encouragingly, a BLAST search of the recently released Pacific Biosciences Ler-0 genome sequence (available at http://www.pacb.com/devnet/) that was obtained by *de novo* assembly using a novel HGAP algorithm with improved ability to resolve long repeats [39], revealed the presence of a complete duplicated *SEC10* locus, with hits of over 98% nucleotide sequence identity covering 97% of the 18-kb sequence segment we submitted to ENA, independently confirming our observations.

We confirmed that both *SEC10* isoforms are expressed, as already indicated by existing cDNA sequences corresponding to both versions, albeit both the public sequence data and our observations document higher expression of the *SEC10b* paralog. Publicly available microarray data from the Genevestigator database, obtained using the ATH1 Affymetrix DNA chip show constitutive *SEC10* expression in all *Arabidopsis* tissues and stages [40]. Nevertheless, the specificity of eleven 25-bp-probes (245211_at) on the ATH1 chip referring to the “single-copy” *SEC10* gene is uncertain, because they probably recognize both *SEC10* paralogs – all probes have full identity to *SEC10a*, whereas seven probes match fully and four probes match each with one mismatch the *SEC10b* sequence. Thus, analyses of promoter specificity and protein localization using reporter genes will be necessary to investigate paralog-specific *SEC10* expression patterns. Without such data, covering preferentially multiple ecotypes or species, we can only speculate whether the apparent underrepresentation of the *SEC10a* transcript under standard culture conditions reflects distinct environmental regulation of the two copies, restriction of the *SEC10a* expression to some minority cell type(s), or even ongoing pseudogenization of this paralog.

The lack of observable phenotypic deviations in single mutants favors a hypothesis that the two genes exhibit mostly overlapping expression patterns in *Arabidopsis* tissues and are to a large extent functionally redundant, although *SEC10b* is apparently more abundantly expressed based on our semi-quantitative RT-PCR experiments with paralog-specific primers (Figure 3), as well as on the failure to amplify the *SEC10a* transcripts in RT-PCR with paralog-indiscriminating primers. A similar situation has been documented for *SEC5a* and *SEC5b* duplicated genes, coding for another exocyst subunit, where *SEC5a* has considerably higher expression than *SEC5b*, although otherwise sharing a similar expression pattern (data from Genevestigator; [40]). The expression of *SEC5b* only in *sec5a* mutants is sufficient for cellular functions, and a mutant phenotype is apparent only in *sec5a sec5b* double mutants, which could be obtained by recombination, given that the two *SEC5* copies reside on different chromosome arms [29]. However, in the case of the tandemly arranged *SEC10a* and *SEC10b*, double mutants would be extremely difficult to obtain, due to the extremely restricted space for recombination between the two genes. Our analysis of *SEC10a/SEC10b* expression in whole seedlings and three entire organs, however, does not exclude the possibility of paralog-specific expression patterns in particular cell types or tissues. Such differences in paralog expression has been indeed found for several pairs of duplicated genes encoding exocyst subunits – e.g. *SEC15a* and *SEC15b*, *EXO70A1* and *EXO70A2*, or *EXO70H3* and *EXO70H4* (data from Genevestigator [22], [40]).

The likely functional redundancy of *SEC10a* and *SEC10b* is supported also by comparing the protein sequences of the two paralogs. An alignment of SEC10a and SEC10b protein sequences showed substitutions of four amino acid residues and an indel four amino acid residues long (Figure S4). Three of the substitutions are more or less synonymous with respect to their biochemical and sterical properties and we suppose no major difference in the structure of the two SEC10 proteins. The fourth substitution, T679P, was considered potentially more consequential because a proline substitution could conceivably disrupt an α-helical structure, which is dominant and functionally essential in all exocyst subunits [41]–[43]. However, no α-helix is predicted (using Jalview 2.8; http://www.jalview.org/) in this region or in the indel region of SEC10.

In yeast and metazoans, major phenotypic defects resulting from affected vesicle trafficking have been found for overexpression of dominant-negative variants, deletion or knockdown of the Sec10 exocyst subunit [44]–[46]. It is likely that the SEC10 exocyst subunit is essential in *Arabidopsis* and total loss of the SEC10 function would cause a severe phenotypic deviation in *Arabidopsis* as well, similarly to loss-of-function exocyst mutants in SEC6 and SEC8 (both encoded by single genes) and a double mutant in SEC5a SEC5b that all exhibit a complete pollen-transmission defect due to impaired pollen tube germination and growth [29], [31]. Since generation of *sec10a sec10b* double mutants in *Arabidopsis* by crossing is impractical due to extremely tight genetic linkage, further experiments, including a knockdown of both *SEC10* genes are required to test this hypothesis. A strategy employing inducible RNAi expression would have to be used in plants with careful characterization of the efficiency of an inhibitory construct, bearing also in mind the expected essential role of the SEC10 exocyst subunit. So far, the lack of observable phenotypic deviations in mutants for either copy of *sec10*, together with minimal differences in protein sequences of SEC10a and SEC10b, point to functional redundancy of these duplicated genes. It would be interesting to reinvestigate the actual number of gene copies in other cases of knock-outs of “single-copy” genes with a surprising lack of a mutant phenotype.

When did the *SEC10* duplication occur in evolution? We found that the duplication is present in at least three different ecotypes of *A. thaliana*, but we could not experimentally detect any *SEC10* tandem duplication in *A. lyrata*. In addition, *A. thaliana SEC10a* and *SEC10b* are mutually more similar than any of them to the *A. lyrata SEC10* (Data S2). This indicates that the duplication most likely occurred after the divergence of the two *Arabidopsis* species. Experimental examination of additional *Arabidopsis* species and other genera of the Brassicaceae family is, however, necessary to make such a statement robust. Nevertheless, the apparently recent origin of the *SEC10* duplication may suggest that it might not yet passed the fixation stage [2], and that it perhaps may not be stable in the long term. Indeed, genes encoding interaction-rich proteins, such as subunits of highly interconnected protein complexes, tend to tolerate tandem duplications rather poorly [7].

Although the observed duplication of a functional gene conflicts with the balance hypothesis, which proposes that single-gene duplication of genes coding for the subunits of protein complexes should be deleterious [47], it is compatible with the hypothesis that duplicated genes provide genetic robustness against null mutations [48], as well as with the possibility that subtle subfunctionalization of duplicated genes may contribute to robustness towards “epigenetic load” [49], especially in sessile organisms. Single knockout data from 5360 *A. thaliana* lines indicate that duplicated genes play a significant role in functional compensation, where duplications tend to persist for a longer time in case of a more severe phenotype of single knock-outs than in the case of a less severe phenotype [50].

## Conclusions

To summarize, we report here a hitherto undocumented *A. thaliana* gene duplication that has resulted in the omission of a functional, expressed gene from the reference genome sequence, due to a sequence assembly error. Similarly to mammalian genomes, some nearly identical gene duplications remain hidden in the current reference sequence of a presumably well-characterized genome of *Arabidopsis thaliana* (and possibly other genomes), and such errors may even propagate in sequencing of new *Arabidopsis* accessions or related species. Since the evidence presented in this paper does not currently meet all the criteria for *A. thaliana* reference sequence update, as stated in the corresponding TAIR policy and as employed in the genome maintenance and (re)annotation process (see http://Arabidopsis.org/doc/portals/genAnnotation/gene_structural_annotation/ref_genome_sequence/11413 and [8]), we would like to encourage researchers responsible for the *A. thaliana* genome sequencing to perform an independent re-sequencing of the *SEC10* locus and update the reference genome sequence of *A. thaliana*. In addition, if original sequence reads are still available, re-assembling of the whole genome sequence from original sequence reads using up-to-date approaches would be advisable to reveal possible similar instances of missed genes.

## Materials and Methods

### Plants, cultivation and genotyping

*A. thaliana* Columbia-0 (Col-0) plants were used for all experiments unless stated otherwise. Landsberg erecta (Ler-0) and Nossen (No-0) ecotypes, together with *Arabidopsis lyrata* (Magnus Nordborg, GMI, Vienna), were also included for an analysis of the *SEC10* gene duplication.

T-DNA insertion mutant lines are listed in Table S1, Table 1 and Figure 4. Seeds were obtained from either NASC [51] or GABI-Kat [52]. Each T-DNA line was backcrossed to Col-0. Seeds were first surface sterilized (70% ethanol for 3 min, 10% commercial bleach for 10 min, washing three times in sterile distilled water) and vernalized for 3 days. Plants were grown in a growth chamber at 21°C and 16 h light per day – first 10 days on vertical agar plates with half-strength Murashige and Skoog medium (Duchefa Biochemie), and then in turf tablets (Jiffy Products International, Norway).

Plants were genotyped using PCR with T-DNA-specific primers (SALK_LBb1, GABI_o8760 or SAIL_LB3) and *SEC10*-specific primers; for primer combinations and sequences see Figure 4A and Table S3. DNA was extracted from 20 mg of fresh leaves from one-month-old plants [53]. Products of PCR genotyping were sequenced using a primer specific to the T-DNA left border (LBb1 for SALK, o8760 for GABI, or LB3 for SAIL lines) to determine in which *SEC10* copy the T-DNA is located and where it is positioned within the gene.

### *SEC10* locus mapping, cloning and sequencing

To confirm the presence and orientation of a tandem *SEC10* duplication, primers A and B or A_lyrata and B_lyrata (Figure 1 and Table S3) were used for PCR reactions on genomic DNA from *A. thaliana* or *A. lyrata*, respectively; genomic DNA was extracted as described above.

The Phusion High-Fidelity DNA Polymerase (New England BioLabs) was employed for amplification of four overlapping segments of the *SEC10* locus. Pairs of primers and annealing temperatures were as follows: Middle_fw + At5g12380 58°C (a-I), IG_fw + Middle_rv 53°C (a-II), Middle_fw + IG_rv 61°C (b-I), At5g12360 + Middle_rv 53°C (b-II) (Figure 2); for primer sequences see Table S3. PCR products were extracted from the agarose gel and cloned into the pJET1.2 blunt cloning vector using the CloneJET PCR Cloning Kit (Fermentas) following the Blunt-End Cloning protocol.

Two clones of each construct were sequenced using the BigDye Terminator v3.1 Cycle Sequencing Kit (Applied Biosystems). Primers for sequencing were designed on the basis of available cDNA sequences (AF479280 and AK222187) to match both *SEC10* genes (Table S2 and S3). pJET1.2 forward and reverse sequencing primers from the CloneJET PCR Cloning Kit were also used for sequencing. Reads covering the whole *SEC10* locus were assembled using the MACAW software [54], [55]. The revised locus sequence was deposited in the ENA database [HG764169].

### Cloning of *SEC10a* and *SEC10b* cDNA

The clone APZL19f10R, represented by the EST sequence GenBank: AV528809, was identified by BLAST (http://blast.ncbi.nlm.nih.gov/) as the *SEC10* cDNA clone with the longest 5′ UTRs preceding the predicted coding sequence, and was therefore selected for complete sequencing. The clone was obtained from the Kazusa DNA Research Institute (http://est.kazusa.or.jp/en/plant/arabi/EST/), subcloned, and sequenced. The assembled sequence was trimmed for vector sequences and deposited in GenBank with the accession number AF479280. The gene corresponding to this cDNA is now called *SEC10b*.

The coding sequence of the *SEC10b* cDNA was also amplified and cloned from total cDNA prepared from 100 mg of Col-0 7-day-old seedlings using the RNeasy Plant Mini Kit (Qiagen) followed by RT-PCR using the Transcriptor High Fidelity cDNA Synthesis Kit (Roche) according to manufactureŕs instructions. The coding sequence of the *SEC10a* cDNA was cloned analogically, but from total cDNA prepared from homozygous *sec10b* mutants (SALK_120710). Cloning primers, S10-Start and S10-Stop (Table S3), matching both *SEC10* copies and starting at the start and stop codons, respectively, were used. PCR products were cloned into the pJET1.2/blunt cloning vector (Fermentas). For analysis of the *SEC10* identity, the coding sequence of *SEC10* was amplified from each clone tested and digested by the *Bpi*I restrictase (Fermentas) that allows to discriminate between *SEC10a* and *SEC10b* (Figure S4B). *Bpi*I cuts *SEC10b* (2490 bp total length) at four positions (78, 392, 681 and 1867 bp), while *SEC10a* (2478 bp) at three positions only (78, 392 and 681 bp).

### Expression analysis in tissues and mutant plants

To analyze the expression level of *SEC10a* and *SEC10b*, total cDNA was prepared from 100 mg of young seedlings (7-day-old), roots (14-day-old), true leaves and flowers (both from one-month-old plants) using the RNeasy Plant Mini Kit (Qiagen) followed by the RT-PCR Transcriptor High Fidelity cDNA Synthesis Kit (Roche). RNA concentration was measured using NanoDrop 1000 (Thermo Scientific). Plasmid clones pda16746 and pda07158 carrying AK222187 and AY096638 (*Arabidopsis* full-length cDNA developed by the plant genome project of RIKEN Genomic Sciences Center [30], [56]), respectively, were used as controls for specific amplification of each *SEC10* copy. Semi-quantitative PCR was performed using the DreamTaq polymerase (Thermo Scientific) and S10-Start primer matching both *SEC10* copies and S10a-3UTR or S10b-3UTR primers matching specifically *SEC10a* or *SEC10b*, respectively (Table S3). Actin-specific primers (ACT7-fw and ACT7-rv; Table S3) were used as a control of the temple concentration. Annealing temperatures used in PCR reactions were 59°C for *SEC10a*, 62°C for *SEC10b,* and 62°C for *ACT7*. Number of PCR cycles was 32 for *SEC10* genes, 34 for controls and 25 for *ACT7*.

## Supporting Information

Figure S1 **Tandem duplication of the** ***SEC10*** **gene in other** ***Arabidopsis*** **accessions.**
(PDF)

Figure S2 **Dot plot of the** ***SEC10*** **locus.**
(PDF)

Figure S3 **Alignment of predicted SEC10a and SEC10b protein sequences.**
(PDF)

Figure S4 **Analysis of** ***SEC10*** **expression in** ***A. thaliana*** **.**
(PDF)

Table S1 **Segregation ratio of** ***sec10*** **mutant lines.**
(PDF)

Table S2 **Assignment of previously published** ***SEC10*** **cDNA sequences to the two** ***SEC10*** **paralogs.**
(PDF)

Table S3 **List of primers used in this study.**
(PDF)

Data S1 **Alignment of** ***SEC10a*** **and** ***SEC10b*** **genes including their flanking sequences.** Sequences of *SEC10a* and *SEC10b* genes from the revisited *SEC10* locus [EMBL: HG764169] were aligned using Clustal X2.1 [57]. Exon-intron borders were manually corrected. The file can be opened in Clustal W, BioEdit, Jalview or similar software.
(ALN)

Data S2 **Alignment of** ***A. thaliana SEC10a*** **and** ***SEC10b*** **genes with the** ***A. lyrata SEC10*** **gene.** Sequences of *A. thaliana SEC10a* and *SEC10b* genes from the revisited *SEC10* locus [EMBL: HG764169] and the sole *A. lyrata SEC10* gene (GenBank: ADBK01001175.1, nucleotides 173660-167031, complementary strand) were aligned using Clustal X2.1 [57]. The file can be opened in Clustal W, BioEdit, Jalview or similar software.
(ALN)
