# Supplementary material for: Dissecting a Hidden Gene Duplication: The Arabidopsis thaliana SEC10 Locus
Source: PLoS One. 2014 Apr 11;9(4):e94077. doi: 10.1371/journal.pone.0094077 (PMC3984084; doi:10.1371/journal.pone.0094077)
Supplement: Figure S1 — Tandem duplication of the SEC10 gene in other Arabidopsis accessions. (PDF) [file pone.0094077.s001.pdf]

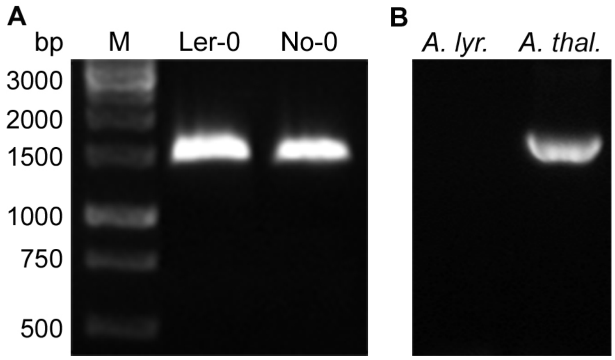

Figure S1. Tandem duplication of the *SEC10* gene in other *Arabidopsis* accessions.

(A) Products of PCR reactions with outward-facing A and B primers (see Figure 1) on genomic DNA templates from Landsberg erecta (Ler-0) and Nossen (No-0) ecotypes of *A. thaliana*.
(B) Products of PCR reactions using *Arabidopsis lyrata*-specific variants of the A and B primers on genomic DNA templates from *A. lyrata* or *A. thaliana* (Col-0), respectively. Although these primers were designed according to the *A. lyrata* sequence, they can recognize the *A. thaliana* template, providing a kind of a positive control.
