# Supplementary material for: Dissecting a Hidden Gene Duplication: The Arabidopsis thaliana SEC10 Locus
Source: PLoS One. 2014 Apr 11;9(4):e94077. doi: 10.1371/journal.pone.0094077 (PMC3984084; doi:10.1371/journal.pone.0094077)
Supplement: Figure S2 — Dot plot of the SEC10 locus. (PDF) [file pone.0094077.s002.pdf]

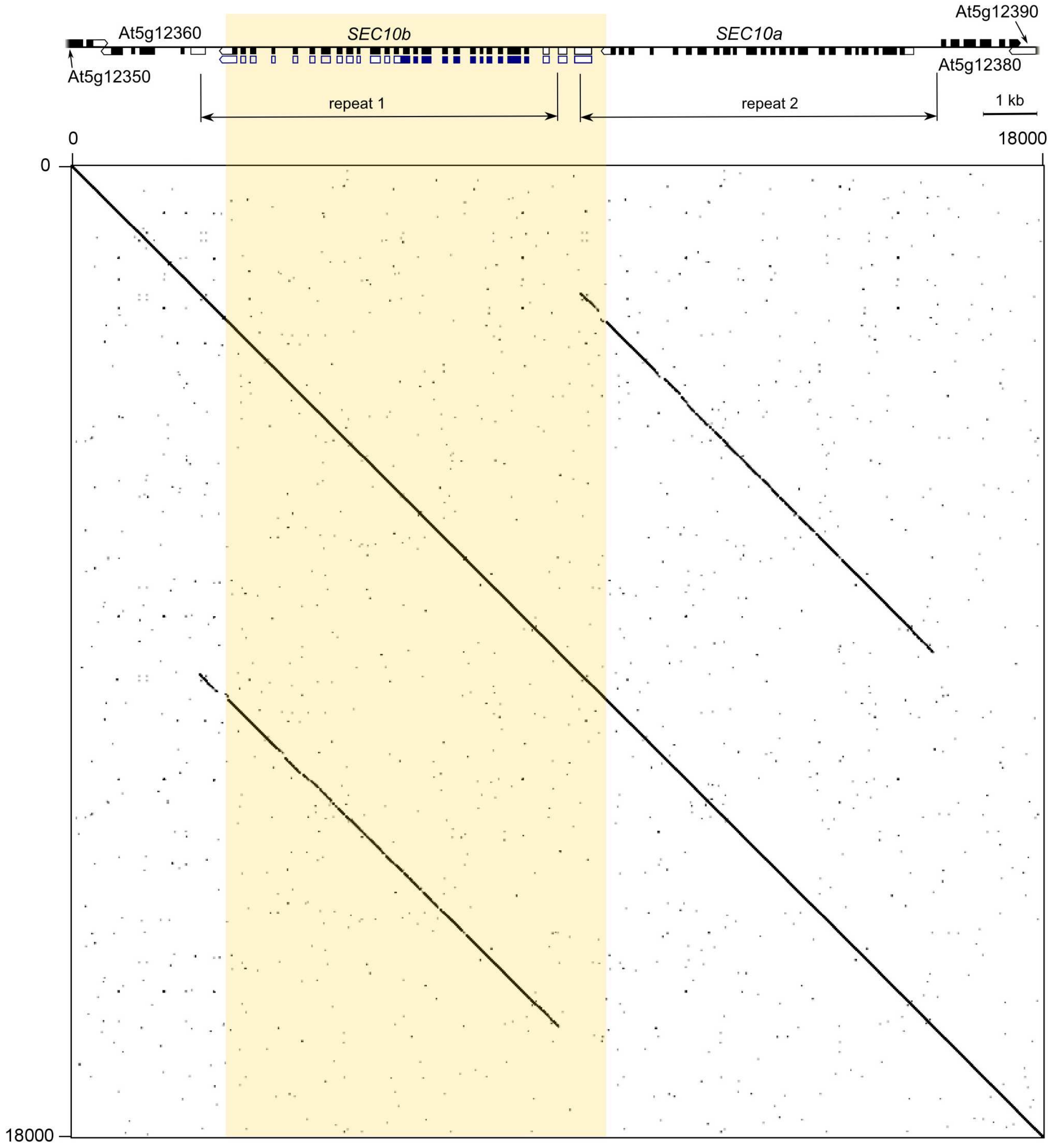

Figure S2. Dot plot of the *SEC10* locus.

A dot plot of the whole assembled *SEC10* locus and neighboring genes on the chromosome 5 against itself (based on the 18-kbp long revisited sequence HG764169). The orange strip marks the region omitted from the *A. thaliana* reference genome sequence. Coding exons (or their parts) are shown as filled boxes, non-coding exons (or their parts) as empty boxes; for *SEC10b*, two versions of experimentally supported cDNA are shown, albeit one of them may be a product of aberrant splicing (in blue, corresponding to the AK318699 cDNA). The plot was produced using Gepard [ref. bellow].

Reference: Krumsiek J, Arnold R, Rattei T (2007) Gepard: a rapid and sensitive tool for creating dotplots on genome scale. Bioinformatics 23: 1026-1028.
