# Supplementary material for: Dissecting a Hidden Gene Duplication: The Arabidopsis thaliana SEC10 Locus
Source: PLoS One. 2014 Apr 11;9(4):e94077. doi: 10.1371/journal.pone.0094077 (PMC3984084; doi:10.1371/journal.pone.0094077)
Supplement: Figure S3 — Alignment of predicted SEC10a and SEC10b protein sequences. (PDF) [file pone.0094077.s003.pdf]

```
NP_568270.3    1 MTEGIRARGPRSSSVNSVPLILDIEDFKGDFSFDALFGNLVNDLLPSFLDEEADSGDGHG 60
AK222187       1 MTEGIRARGPRSSSVNSVPLILDIEDFKGDFSFDALFGNLVNDLLPSFLDEEADSGDGHG 60
AF479280       1 MTERIRARGPRSSSVNSVPLILDIEDFKGDFSFDALFGNLVNDLLPSFLDEEADSGDGHG 60

NP_568270.3   61 NIAGVDGLTNGHLRGQSAPLSSAPFFPEVDGLLSLFKDACKELVDLRKQVDGRLNTLKKE 120
AK222187      61 NIAGVDGLTNGHLRGQSAPLSSAPFFPEVDGLLSLFKDACKELVDLRKQVDGRLNTLKKE 120
AF479280      61 NIAGVDGLTNGHLRGQSAPLSSAPFFPEVDGLLSLFKDACKELVDLRKQVDGRLNTLKKE 120

NP_568270.3  121 VSTQDSKHRKTLTEIEKGVDGLFESFARLDGRISSVGQTAAKIGDHLQSADAQRETASQT 180
AK222187     121 VSTQDSKHRKTLTEIEKGVDGLFESFARLDGRISSVGQTAAKIGDHLQSADAQRETASQT 180
AF479280     121 VSTQDSKHRKTLTEIEKGVDGLFESFARLDGRISSVGQTAAKIGDHLQSADAQRETASQT 180

NP_568270.3  181 IDLIKYLMEFNGSPGDLMELSALFSDDSRVAEAASIAQKLRSFAEEDIGRQGASAAAGNA 240
AK222187     181 IDLIKYLMEFNGSPGDLMELSALFSDDSRVAEAASIAQKLRSFAEEDIGRQGASAAAGNA 240
AF479280     181 IDLIKYLMEFNGSPGDLMELSALFSDDSRVAEAASIAQKLRSFAEEDIGRQGASTAAGNA 240

NP_568270.3  241 TPGRGLEVAVANLQDYCNELENRLLSRFDAASQRRDLSTMSECAKILSQFNRGTSAMQHY 300
AK222187     241 TPGRGLEVAVANLQDYCNELENRLLSRFDAASQRRDLSTMSECAKILSQFNRGTSAMQHY 300
AF479280     241 TPGRGLEVAVANLQDYCNELENRLLSRFDAASQRRDLSTMSECAKILSQFNRGTSAMQHY 300

NP_568270.3  301 VATRPMFIDVEVMNSDIRLVLGDHGSQPSPSNVARGLSALFKEITDTVRKEAATITAVFP 360
AK222187     301 VATRPMFIDVEVMNSDIRLVLGDHGSQPSPSNVARGLSALFKEITDTVRKEAATITAVFP 360
AF479280     301 VATRPMFIDVEVMNSDIRLVLGDHGSQPSPSNVARGLSALFKEITDTVRKEAATITAVFP 360

NP_568270.3  361 TPNEVMAILVQRVLEQRVTGILDKILAKPSLMSPPPVQEGGLLLYLRMLAVAYERTQELA 420
AK222187     361 TPNEVMAILVQRVLEQRVTGILDKILAKPSLMSPPPVQEGGLLLYLRMLAVAYERTQELA 420
AF479280     361 TPNEVMAILVQRVLEQRVTGILDKILAKPSLMSPPPVQEGGLLLYLRMLAVAYERTQELA 420

NP_568270.3  421 KDLRAVGCGDLDVEDLTESLFSSHKDEYPEHERASLKQLYQAKMEELRAESQQVSESSGT 480
AK222187     421 KDLRAVGCGDLDVEDLTESLFSSHKDEYPEHERASLKQLYQAKMEELRAESQQVSESSGT 480
AF479280     421 KDLRAVGCGDLDVEDLTESLFSSHKDEYPEHERASLKQLYQAKMEELRAESQQVSESSGT 480

NP_568270.3  481 IGRSKGASISSSLQQISVTVVTDFVRWNEEAITRCTLFSSQPATLAANVKAIFTCLLDQV 540
AK222187     481 IGRSKGASISSSLQQISVTVVTDFVRWNEEAITRCTLFSSQPATLAANVKAIFTCLLDQV 540
AF479280     481 IGRSKGASISSSLQQISVTFVTEFVRWNEEAITRCTLFSSQPATLAANVKAIFTCLLDQV 540

NP_568270.3  541 SVYITEGLERARDSLSEAAALRERFVLG----RRVAAAAASAAEAAAAAGESSFKSFMVA 596
AK222187     541 SVYITEGLERARDSLSEAAALRERFVLG----RRVAAAAASAAEAAAAAGESSFKSFMVA 596
AF479280     541 SVYITEGLERARDSLSEAAALRERFVLGTSVSRRVAAAAASAAEAAAAAGESSFKSFMVA 600

NP_568270.3  597 VQRCGSSVAIVQQYFANSISRLLLPVDGAHAASCEEMSTALSKAEAAAYKGLQQCIETVM 656
AK222187     597 VQRCGSSVAIVQQYFANSISRLLLPVDGAHAASCEEMSTALSKAEAAAYKGLQQCIETVM 656
AF479280     601 VQRCGSSVAIVQQYFANSISRLLLPVDGAHAASCEEMSTALSKAEAAAYKGLQQCIETVM 660

NP_568270.3  657 AEVDRLLSSEQKSTDYRSTDDGIASDHRPTNACIRVVAYLSRVLESAFTALEGLNKQAFL 716
AK222187     657 AEVDRLLSSEQKSTDYRSTDDGIASDHRPTNACIRVVAYLSRVLESAFTALEGLNKQAFL 716
AF479280     661 AEVDRLLSSEQKSTDYRSPDDGIASDHRPTNACIRVVAYLSRVLESAFTALEGLNKQAFL 720

NP_568270.3  717 TELGNRLEKLLLTHWQKFTFNPSGGLRLKRDLNEYVGFVKSFGAPSVDEKFELLGIIANV 776
AK222187     717 TELGNRLEKLLLTHWQKFTFNPSGGLRLKRDLNEYVGFVKSFGAPSVDEKFELLGIIANV 776
AF479280     721 TELGNRLEKLLLTHWQKFTFNPSGGLRLKRDLNEYVGFVKSFGAPSVDEKFELLGIIANV 780

NP_568270.3  777 FIVAPDSLPTLFEGSPSIRKDAQRFIQLREDYKSAKLATKLSSLWPSLS            825
AK222187     777 FIVAPDSLPTLFEGSPSIRKDAQRFIQLREDYKSAKLATKLSSLWPSLS            825
AF479280     781 FIVAPDSLPTLFEGSPSIRKDAQRFIQLREDYKSAKLATKLSSLWPSLS            829
```

Figure S3. Alignment of predicted SEC10a and SEC10b protein sequences.

An alignment of predicted protein sequences of the AK222187 cDNA (*SEC10a*), AF479280 cDNA (*SEC10b*), and a conceptual protein NP_568270.3 based on the reference cDNA sequence NM_121275.4, corresponding to *SEC10a* by its coding sequence.
