# Supplementary material for: Dissecting a Hidden Gene Duplication: The Arabidopsis thaliana SEC10 Locus
Source: PLoS One. 2014 Apr 11;9(4):e94077. doi: 10.1371/journal.pone.0094077 (PMC3984084; doi:10.1371/journal.pone.0094077)
Supplement: Figure S4 — Analysis of SEC10 expression in A. thaliana . (PDF) [file pone.0094077.s004.pdf]

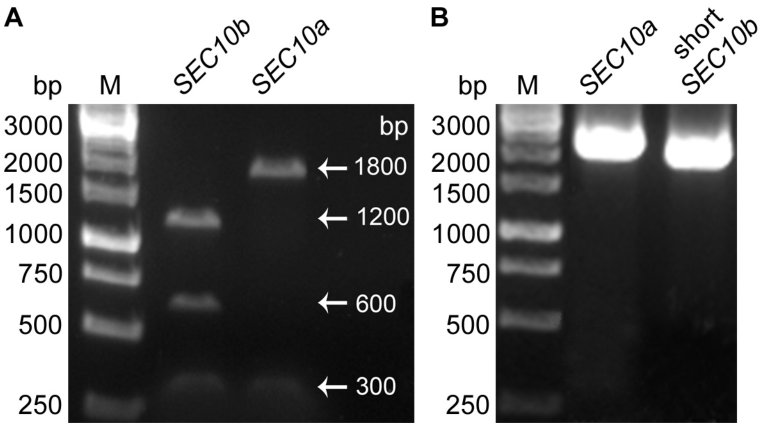

Figure S4. Analysis of *SEC10* expression in *A. thaliana*.

(A) Restriction patterns of *SEC10a* and *SEC10b* coding sequences after digestion with the *Bpi*I restrictase. This restriction analysis was used to screen cloned amplicons obtained with paralog-indisriminating primers.
(B) Two different types of PCR products were obtained after amplification of the *SEC10* coding sequence using paralog-indiscriminating primers and total cDNA extracted from the *sec10b-1* mutant as a template. The longer product corresponds to *SEC10a* and the shorter one to a shortened out-of-frame *SEC10b* version.
