# Supplementary material for: Dissecting a Hidden Gene Duplication: The Arabidopsis thaliana SEC10 Locus
Source: PLoS One. 2014 Apr 11;9(4):e94077. doi: 10.1371/journal.pone.0094077 (PMC3984084; doi:10.1371/journal.pone.0094077)
Supplement: Table S1 — Segregation ratio of sec10 mutant lines. (PDF) [file pone.0094077.s005.pdf]

Table S1. Segregation ratio of *sec10* mutant lines.

Putative heterozygous lines	Genotype by PCR genotyping			No. of plants	Percent. of w/m	Testing ratio w/w : w/m by Chí-square test		Current conclusion	T-DNA position in *SEC10*
	w/w	w/m	m/m			1:2	1:3		
SALK_120710 #1	9	38	0	47	81%	P = 0.031	P = 0.316	normal segregation	*b*
SALK_120710 #2	0	17	0	17	100%	-	-	homozygous line	*b*
SALK_101637 #1	17	56	0	73	77%	P = 0.081	P = 0.786	normal segregation	*b*
SALK_101637 #2	5	19	0	24	79%	P = 0.194	P = 0.637	normal segregation	*b*
SALK_010685 #1	0	48	0	48	100%	-	-	homozygous line	*b*
SALK_010685 #2	0	62	0	62	100%	-	-	homozygous line	*b*
SALK_052001 #1	5	19	0	24	79%	P = 0.194	P = 0.637	normal segregation	*a*
SALK_052001 #2	0	6	0	6	100%	-	-	homozygous line	*a*
SALK_036501	0	20	0	20	100%	-	-	homozygous line	*b*
GABI_302_H05	15	48	0	63	76%	P = 0.109	P = 0.772	normal segregation	*a*
GABI_381_H05	14	46	0	60	77%	P = 0.100	P = 0.766	normal segregation	*a*
GABI_770_C01	16	43	0	59	73%	P = 0.271	P = 0.765	normal segregation	*b*
SAIL_222_B02 #1	12	36	0	48	75%	P = 0.221	P = 1.000	normal segregation	*b*
SAIL_222_B02 #2	0	24	0	24	100%	-	-	homozygous line	*b*

Segregation of multiple putative heterozygous plants of several T-DNA insertional mutant lines was analyzed by PCR. The ratio of wild-type (w/w) and heterozygous (w/m) plants was tested for corresponding to either to 1:2 or 1:3. The latter ratio suggests that heterozygous plants were in fact a mixture of genuine heterozygotes and homozygotes that exhibited a wild-type signal from another *SEC10* paralog in PCR genotyping. Analysis of the T-DNA positions by sequencing from their Left borders determined the T-DNA location in each mutant line in either *SEC10a* or *SEC10b*.
