# Supplementary material for: Dissecting a Hidden Gene Duplication: The Arabidopsis thaliana SEC10 Locus
Source: PLoS One. 2014 Apr 11;9(4):e94077. doi: 10.1371/journal.pone.0094077 (PMC3984084; doi:10.1371/journal.pone.0094077)
Supplement: Table S2 — Assignment of previously published SEC10 cDNA sequences to the two SEC10 paralogs. (PDF) [file pone.0094077.s006.pdf]

Table S2. Assignment of previously published *SEC10* cDNA sequences to the two *SEC10* paralogs.

Accession	Database	Definition	Source	*SEC10* copy
AF479280	GenBank	mRNA, complete CDS	experimental; this work	b
AV528809	GenBank	mRNA, EST	experimental	b
NM_121275.4	GenBank	mRNA, complete CDS	prediction	a
NM_001036794.1	GenBank	mRNA, complete CDS	prediction	a
NM_001036795.2	GenBank	mRNA, complete CDS	prediction	a
AY096638	GenBank	mRNA, complete CDS	experimental	b
AK222187	DDBJ	mRNA, complete CDS	experimental	a
