# Supplementary material for: Dissecting a Hidden Gene Duplication: The Arabidopsis thaliana SEC10 Locus
Source: PLoS One. 2014 Apr 11;9(4):e94077. doi: 10.1371/journal.pone.0094077 (PMC3984084; doi:10.1371/journal.pone.0094077)
Supplement: Table S3 — List of primers used in this study. (PDF) [file pone.0094077.s007.pdf]

Table S3. List of primers used in this study.

Primer	Sequence	Orientation	Purpose
S10-Start	5'-TCAGCTCAAGCTTGGCCACAA-3'	forward	cloning, expression
S10-Stop	5'-ATGACAGAACGAATCAGAGCAAGA-3'	reverse	cloning
A	5'-TGATCTGGGTCCTCTTGCT-3'	forward	duplication proof
B	5'-ACTGAGGGAGGACTATAAGA-3'	reverse	duplication proof
A_lyrata	5'-TGCTCTGATTCCTTCTGTCATC-3'	forward	duplication proof
B_lyrata	5'-ACTTCGGGAGGACTATAAGAGT-3'	reverse	duplication proof
At5g12360	5'-CCAATGCATTCTCGTCCTGA-3'	forward	cloning
At5g12380	5'-CAAGCCGCCTTGATGTTTTC-3'	reverse	cloning
IG_Fw	5'-CGAACAGATAGATAGAGAGAG-3'	forward	cloning
IG_Rv	5'-AAACATCATTGAGCATATTTGACA-3'	reverse	cloning
Middle_Fw	5'-ACTCTGAGACTTGCTGACTC-3'	forward	cloning
Middle_Rv	5'-GCATATGAGAGAACACAAGAACT-3'	reverse	cloning
S10b-1_com	5'-AATAGTAAACTTTCATTGTCGTGGAA-3'	forward	genotyping
S10a-1/2_com	5'-TTTATGTATATGACTTTGCCGTTATTT-3'	reverse	genotyping
S10a-1/2_wt	5'-CCTCAGCAAAGGATCCTGATATC-3'	forward	genotyping
S10b-2_com	5'-CACTCTTCTGCTAACGCTTGTTC-3'	reverse	genotyping
S10b-2_wt	5'-GTCATTTTTTTCTCCTGATTCTGAG-3'	forward	genotyping
GABI_ o8760	5'-GGGCTACACTGAATTGGTAGCTC-3'	-	genotyping
SAIL_LB3	5'-CATCTGAATTTCATAACCAATCTCG-3'	-	genotyping
SALK_LB	5'-GAACAACACTCAACCCTATCTCGGGC-3'	-	genotyping
SeqA	5'-TGCGGCCGCAAGCTTGGCCACAA-3'	forward	sequencing
SeqB	5'-GTCCAACAAATGCCTGCATA-3'	reverse	sequencing
SeqC	5'-TAAGCAGCTGCCTCAGCTTTGG-3'	forward	sequencing
SeqD	5'-TAGCAATGTTGCCCGTGGA-3'	reverse	sequencing
SeqE	5'-AAGTGTTGGACAGACCGCT-3'	reverse	sequencing
SeqF	5'-TGCCATGGCCATCCCCA-3'	forward	sequencing
SeqG	5'-ACTGGAAGTTGCAGTTGCTA-3'	reverse	sequencing
SeqH	5'-GTTGCGGTAGCAGTGTTG-3'	reverse	sequencing
SeqI	5'-GTTCAGTCAGGAAGGCTTG-3'	forward	sequencing
SeqJ	5'-GTTGCTCCAGATAGTCTGC-3'	reverse	sequencing
SeqK	5'-TCTCAGTCAGTGTCTTCCG-3'	forward	sequencing
S10a-3UTR	5'-GAGAAGTGGAAATTCACAAATATG-3'	forward	expression
S10b-3UTR	5'-TTTTTGGACCAAAACGATATCTCA-3'	forward	expression
ACT7-Fw	5'-TTATGTCGCTCTTGACTACGAGCA-3'	forward	expression
ACT7-Rv	5'-AAGCATTTCCTGTGAACAATCGATG-3'	reverse	expression
